# Invariant natural killer T cells regulate anti-tumor immunity by controlling the population of dendritic cells in tumor and draining lymph nodes

**DOI:** 10.1186/s40425-014-0037-x

**Published:** 2014-10-14

**Authors:** Karsten A Pilones, Joseph Aryankalayil, James S Babb, Sandra Demaria

**Affiliations:** Department of Pathology, New York University School of Medicine, New York, NY 10016 USA; Department of Radiology, New York University School of Medicine, New York, NY 10016 USA; Department of Radiation Oncology, New York University School of Medicine, New York, NY 10016 USA; New York University School of Medicine, Alexandria Center for Life Sciences, 450 East 29th St, Room 324B, New York, NY 10016 USA

**Keywords:** Breast cancer, CD1d, CD137, CTLA-4, CD8^+^ T-cells, Dendritic cells, Immunoregulation, Invariant NKT cells, Radiotherapy

## Abstract

**Background:**

Invariant natural killer T (iNKT) cells are CD1d-restricted T cells, which respond rapidly to antigen recognition and promote development of anti-tumor immunity in many tumor models. Surprisingly, we previously found that mice deficient in iNKT cells developed spontaneous CD8^+^ T cells responses partially effective at inhibiting metastases in mice bearing the 4T1 mammary carcinoma, and showed a markedly improved response to treatment with local radiotherapy and anti-CTLA-4 antibody compared to wild type (WT) mice.

**Methods:**

To understand the mechanisms of the immunosuppressive function of iNKT cells, dendritic cells (DCs) were analyzed by immunohistochemistry and flow cytometry in WT and iNKT-deficient (iNKT^−/−^) mice. The effects of antibody-mediated blockade of CD1d on DC number and phenotype, priming of anti-tumor T cells, and tumor response to treatment with local radiotherapy and anti-CTLA-4 antibody were evaluated. To determine if the improved response to treatment in the absence of iNKT cells was independent from the immunotherapy employed, 4T1-tumor bearing WT and iNKT^−/−^ mice were treated with local radiotherapy in combination with antibody-mediated CD137 co-stimulation.

**Results:**

DCs in 4T1 tumors and tumor-draining lymph nodes but not distant lymph nodes were significantly reduced in WT mice compared to iNKT^−/−^ mice (p < 0.05), suggesting the selective elimination of DCs cross-presenting tumor-associated antigens by iNKT cells. Consistently, priming of T cells to a tumor-specific CD8 T cell epitope in mice treated with radiotherapy and anti-CTLA-4 or anti-CD137 was markedly enhanced in iNKT^−/−^ compared to WT mice. CD1d blockade restored the number of DC in WT mice, improved T cell priming in draining lymph nodes and significantly enhanced response to treatment.

**Conclusions:**

Here we describe a novel mechanism of tumor immune escape mediated by iNKT cells that limit priming of anti-tumor T cells by controlling DC in tumors and draining lymph nodes. These results have important implications for the design of immunotherapies targeting iNKT cells.

**Electronic supplementary material:**

The online version of this article (doi:10.1186/s40425-014-0037-x) contains supplementary material, which is available to authorized users.

## Background

Natural killer T (NKT) cells comprise a subset of lymphocytes originating from a distinct developmental lineage [1] which bridge innate and adaptive immunity and modulate immune responses in autoimmunity, malignancies and infections [2]. Although initially identified by co-expression of conventional αβ T-cell receptors (TCR) and markers typically associated with natural killer (NK) cells [3], NKT are currently distinguished on the basis of CD1d restriction as well as specific usage of TCRα chains [4]. In both mice and humans, most NKT cells express TCRs formed by the rearrangement of a canonical α chain (Vα14 in mice, Vα24 in humans) and a limited set of Vβ chains (Vβ.2, Vβ7, Vβ2 in mice, Vβ11 in humans) and are commonly referred to as type I or invariant natural killer T (iNKT) cells [5,6]. A smaller NKT cell subset utilizes a more diverse set of TCR αβ chains and is referred to as type II or non-invariant NKT cells [7].

Identification of α-galactosylceramide (α-GalCer) as a strong agonist selective for iNKT cells [8] facilitated their characterization using α-GalCer-loaded CD1d tetramers [9]. In several tumor models, iNKT cells were found to perform important immunosurveillance functions and become key effectors of tumor rejection when activated by α-GalCer [10-13]. Expression of high levels of Fas Ligand, perforin, and granzyme B by iNKT cells underlies their cytolytic activity against CD1d^+^ tumor cells [14] and myeloid cells with immunosuppressive function present in the tumor microenvironment [15]. In addition, iNKT cells exert anti-tumor functions by rapid and robust secretion of cytokines that improve DC ability to cross-prime anti-tumor T cells [10,12,16,17] and enhance recruitment of other effectors such as NK cells, CD4^+^ T helper-1 (Th1) and CD8^+^ cytotoxic T (CTL) cells [13,18].

Experimental data in different systems indicate a functional plasticity of iNKT cells. iNKT cells can promote the polarization of adaptive immune responses towards both Th1 and Th2 and can secrete immunosuppressive cytokines [19]. The regulatory function of iNKT cells has been demonstrated in multiple models of autoimmune diseases in which iNKT cells played essential roles in maintenance of tolerance [20-22]. The mechanisms that determine whether iNKT cells act to promote immune activation or tolerance remain incompletely understood, but the inflammatory context in which interactions of iNKT cells with CD1d^+^ myeloid cells take place is thought to be a key factor [23,24].

The 4T1 mouse mammary carcinoma is a model for triple negative breast cancer and shows an aggressive behavior with rapid spread of metastatic cells to the lungs after subcutaneous injection. We previously found that 4T1 tumor-bearing iNKT-deficient (iNKT^−/−^) mice developed a spontaneous CD8^+^ T cell response that was partially effective at controlling metastases in the lungs [25]. Response to treatment with local radiotherapy and anti-CTLA-4 monoclonal antibody (mAb) was also markedly improved in iNKT^−/−^ compared to wild type (WT) mice with half of the mice rejecting completely the primary irradiated tumor and lung metastases and showing long-term survival compared to none of the WT mice [25]. These data implicated iNKT cells as major regulators of the spontaneous as well as immunotherapy-elicited anti-tumor immune response to 4T1 tumor.

CD4^+^ regulatory T cells (Treg) have been shown to limit the development of anti-tumor T cells by killing DCs in tumor-draining lymph nodes (dLN) [26]. DCs are a major subset of CD1d^+^ antigen-presenting cells (APC) that interact with iNKT cells. Therefore, we hypothesized that iNKT cells may be limiting the anti-tumor immune response in 4T1 tumor-bearing mice by controlling the loco-regional population of DCs in the tumor and dLNs. For the first time, we present evidence that WT mice have reduced numbers of DCs in tumor and dLN compared to iNKT^−/−^ mice, and reduced priming of tumor-specific CD8^+^ T cells, as measured by gamma interferon (IFNγ) production in response to a H2-L^d^-restricted peptide derived from a tumor antigen. DC numbers and tumor-specific CD8^+^ T cell responses were at least partially restored by blocking CD1d, resulting in improved response to immunotherapy. Overall, data demonstrate a novel mechanism of immune escape mediated by iNKT cells.

## Results

### Numbers of DCs infiltrating 4T1 tumors and dLN are lower in WT compared to iNKT^−/−^ mice

**Figure 1:**
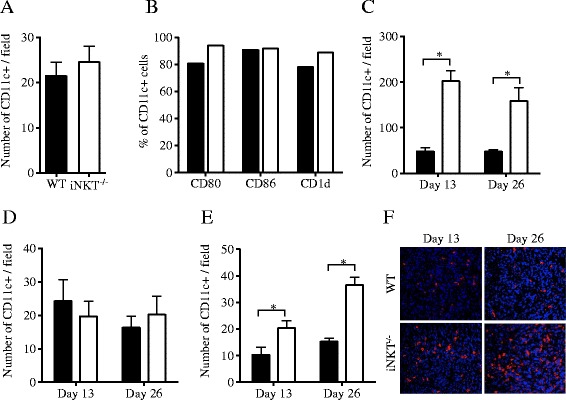
**iNKT cells regulate the number of DCs in 4T1 tumors and draining lymph nodes. (A, B)** Lymph nodes collected from healthy WT (black bars) and iNKT^−/−^ (white bars) mice (N = 3/strain). **(A)** Full-face lymph node sections were stained with anti-CD11c mAb and positive cells counted. **(B)** Dissociated lymph node cells were gated on CD11c + cells and expression of CD1d and co-stimulatory molecules CD80 and CD86 determined by flow cytometry. **(C-F)** WT (black bars) and iNKT^−/−^ (white bars) mice were inoculated s.c. with 4T1 cells. On indicated days, tumor-draining lymph nodes **(C)**, non-draining lymph nodes **(D)**, and tumors **(E, F)** were excised, tissue sections stained with anti-CD11c mAb and positive cells counted. Numbers are the mean ± SD of 3 mice of each strain analyzed at each time point. **(F)** Representative fields (400× magnification). Results are representative of two experiments. ^*^ *p* < 0.05.

We hypothesized that the interaction of iNKT cells with CD1d^+^ APC could modulate the ability of WT mice to develop anti-tumor immune responses upon treatment. To test this hypothesis we compared DCs in healthy and tumor-bearing WT and iNKT^−/−^ mice. iNKT^−/−^ mice were generated by targeted deletion of the Jα18 segment of the T-cell receptor (TCR) and show selective loss of the Vα14Jα18 TCR expressed by iNKT cells while other lymphoid and myeloid populations are not affected [27]. Consistently, there were no differences in the number of DCs present in LNs or their expression of maturation markers CD80 and CD86, including CD1d, between WT and iNKT^−/−^ healthy mice (Figure 1A and B). In contrast, after 4T1 tumor implantation there was a difference in the numbers of DCs in the tumor-draining but not distant LNs (Figure 1C and D), with significantly lower numbers of DCs present in WT compared to iNKT^−/−^ mice. Significantly lower numbers of DCs were also found in the tumors growing in WT compared to iNKT^−/−^ mice (Figure 1E and F). Interestingly, while non-draining LNs of iNKT^−/−^ mice showed a DC density comparable to LNs of healthy mice, DC density was markedly increased in tumor-draining LNs, suggesting that even in the absence of treatment the presence of the growing tumor leads to some activation of the regional immune system. This finding is consistent with the presence of a spontaneous anti-tumor CD8^+^ T cell response in iNKT^−/−^ mice that was partially effective at controlling metastases in the lungs as we have previously reported [25].

Overall, data suggest that in WT mice iNKT cells may control the population of DCs in the tumor and dLN thus precluding the priming of anti-tumor T cells.

### CD1d blockade restores DC numbers and improves tumor-specific T cell cross-priming in WT 4T1 tumor-bearing mice

**Figure 2:**
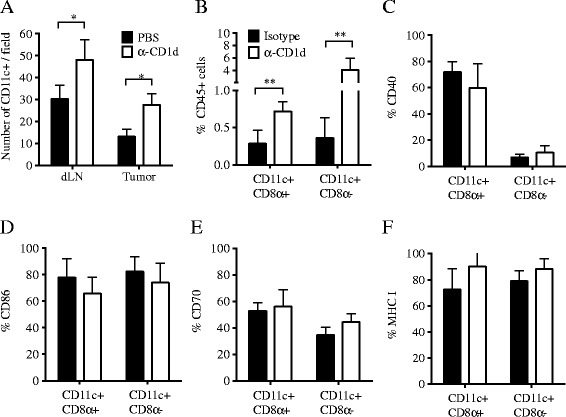
**Blocking CD1d enhances DC numbers in 4T1 tumor-bearing WT mice.** WT mice were inoculated with 4T1 cells on day 0. On days 3, 7 and 11 mice were given either PBS **(A)** or Isotype mAb **(B-F)** (black bars) or anti-CD1d mAb **(A-F)** (white bars) i.p. **(A)** Tumors and dLN were excised on day 13, and tissue sections stained with anti-CD11c mAb. Numbers are the mean ± SD of 3 mice/group. Results are representative of two experiments. **(B-F)** In a separate experiment using the same blocking regimen, tumors were digested for flow cytometry analysis of dissociated cells (N = 9/group). To obtain sufficient material, each sample was prepared by pooling tumors from 3 mice, and 3 independent samples/group analyzed. **(B)** Samples were gated on CD45 + CD11c + cells, and analyzed for expression of **(B)** CD8α, and **(C)** CD40, **(D)** CD86, **(E)** CD70 and **(F)** MHC Class I molecules. ^*^ *p* < 0.05, ^**^ *p* < 0.005.

Elimination of DCs in dLN has been shown to be one mechanism whereby Treg suppress the anti-tumor immune response [26]. To determine if iNKT cells could be responsible for the reduction in the number of DCs in tumors and dLN of WT mice we tested whether inhibiting their interaction with DCs with a mAb that blocks CD1d [28] would lead to increased DC numbers. First, we confirmed that the anti-CD1d mAb used (20H2) was able to inhibit the interaction of the DN32.D3 iNKT-like hybridoma cells with α-GalCer-loaded DCs in vitro in a dose-dependent manner (Additional file 1: Figure S1). In addition, we verified that administration of 20H2 mAb to mice did not cause the depletion of CD1d^+^ cells (Additional file 2: Figure S2) nor induce their stimulation by reverse signaling (Additional file 3: Figure S3). Next, anti-CD1d mAb was given to 4T1 tumor-bearing WT mice starting on day 3 post-tumor inoculation and tumors and dLN analyzed at day 13, a time when tumors are irradiated in treated mice and the availability of DCs that can cross-present the tumor antigens released by radiation is critical [29,30]. CD1d blockade led to a significant increase in the total number of DCs present in tumors and dLNs (Figure 2A). Both immunostimulatory CD8α + and CD8α- DC subsets were increased (Figure 2B), whereas expression of activation markers CD40, CD70, CD86, and of MHC Class I molecules in either subsets of tumor infiltrating DCs were not altered by CD1d blockade (Figure 2C-F). Therefore, data indicate that iNKT cells regulate DC numbers in 4T1 tumor-bearing WT mice via CD1d-mediated interactions.

**Figure 3:**
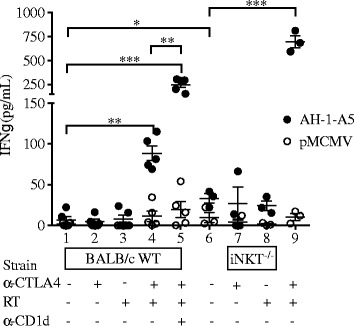
**CD1d blockade enhances IFN-γ response by tumor-specific cells generated by RT + anti-CTLA-4 blockade.** WT or iNKT^−/−^ mice were treated with local tumor radiotherapy in two fractions of 12 Gy given on days 13 and 14 post-tumor inoculation. Mice received anti-CTLA-4 mAb on days 15, 18 and 21. Some mice additionally received anti-CD1d mAb on days 3, 7 and 11 post tumor inoculation. Cells from tumor-draining lymph nodes were collected on day 23 and stimulated with feeder cells pre-loaded with irrelevant peptide pMCMV (open circles) or AH-1-A5 (filled circles). Secreted IFN-γ in the supernatant was measured 48 hours later. Each symbol represents an individual mouse. Bars indicate the mean ± SD of 5 mice/group. ^*^ *p* < 0.05, ^**^ *p* < 0.005, ^***^ *p* < 0.0005.

To determine if blockade of CD1d resulted in improved priming of anti-tumor CD8^+^ T cells in dLN of WT mice treated with local radiotherapy and anti-CTLA-4 mAb [25], we measured tumor antigen-specific production of IFNγ by dLN cells stimulated with a CD8 T cell epitope derived from the tumor antigen gp70 [31]. No IFNγ production was detected in dLNs from WT mice that were untreated or treated with RT or anti-CTLA-4 alone (Figure 3). However, the combination of RT and anti-CTLA-4 induced significant tumor-specific IFNγ production (p < 0.005 versus all other groups), as expected given the therapeutic synergy we have previously shown with this combination [25,29,32]. Importantly, the response was significantly enhanced by blockade of CD1d (mean IFN-γ 248.50 ± 67.41 pg/mL in RT + anti-CTLA-4 + anti-CD1d versus 88.62 ± 19.80 pg/mL in RT + anti-CTLA-4, p < 0.005). Interestingly, tumor antigen-specific IFN-γ production was detected in dLNs from untreated iNKT^−/−^ mice (33.08 ± 10.04 pg/mL for AH1-A5 versus 9.65 ± 10.86 pg/mL for pMCMV, p = 0.05) (Figure 3), a finding consistent with the ability of these mice to inhibit lung metastases in a CD8-dependent manner [25]. This response was not affected by radiotherapy or anti-CTLA-4 as single treatment, but was dramatically increased by their combination (696.12 ± 109.22 pg/mL, p < 0.001 versus control). Remarkably, IFNγ response of dLN cells from mice treated with radiotherapy + anti-CTLA-4 was seven fold higher in iNKT^−/−^ compared to WT mice.

Overall, data strongly support the conclusion that iNKT cells perform a regulatory function and hinder the development of anti-tumor T cells in 4T1 tumor-bearing mice by controlling DC population that cross-present tumor-derived antigens in dLNs.

### CD1d blockade improves response to treatment with radiotherapy and anti-CTLA-4 in WT mice

**Figure 4:**
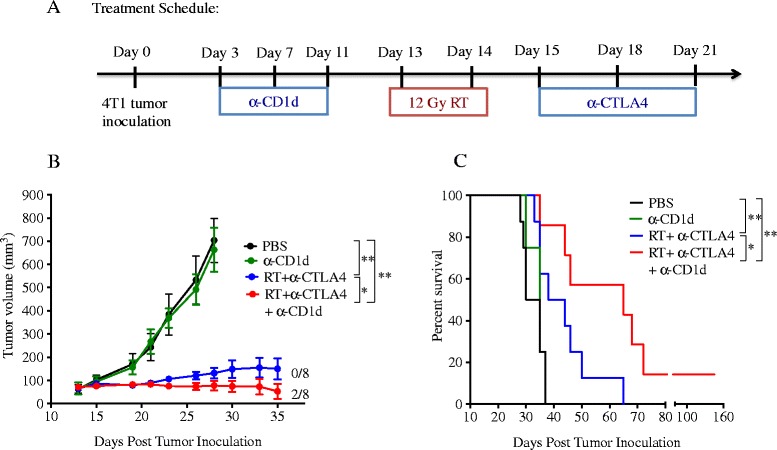
**CD1d blockade improves response of WT mice to treatment with radiotherapy and anti-CTLA-4. (A)** WT 4T1 tumor-bearing mice (N = 8 mice/group) were treated with local tumor radiotherapy in two fractions of 12 Gy given on days 13 and 14 post-tumor inoculation. Mice received anti-CTLA-4 mAb on days 15, 18 and 21. Some mice additionally received anti-CD1d mAb on days 3, 7 and 11 post tumor inoculation. **(B)** Tumor growth is shown for each group until the last day when all mice were alive, day 28 for control and anti-CD1d treated groups, and day 35 for RT + anti-CTLA-4-treated groups. Fractions indicate the number of mice showing complete tumor regression over the total in each group. **(C)** Surviving tumor-free mice were followed until day 150. Data shown are from one of 2 independent experiments performed with similar results. ^*^ *p* < 0.05, ^**^ *p* < 0.005.

**Figure 5:**
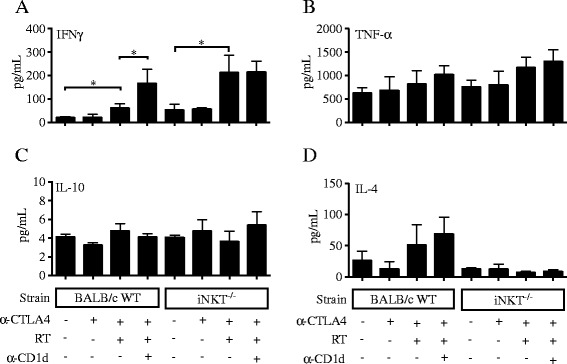
**CD1d blockade enhances intratumoral IFN-γ response induced by RT + anti-CTLA-4 blockade.** WT and iNKT^−/−^ mice were treated with local tumor radiotherapy in two fractions of 12 Gy given on days 13 and 14 post-tumor inoculation. Mice received anti-CTLA-4 mAb on days 15, 18 and 21. Some mice additionally received anti-CD1d mAb or isotype mAb on days 3, 7 and 11 post tumor inoculation. Tumors were harvested at day 22 and concentration of **(A)** IFN-γ, **(B)** TNF-α, **(C)** IL-10 and **(D)** IL-4 were determined. Bars indicate the mean ± SD of 4 mice/group. ^*^ *p* < 0.05.

**Figure 6:**
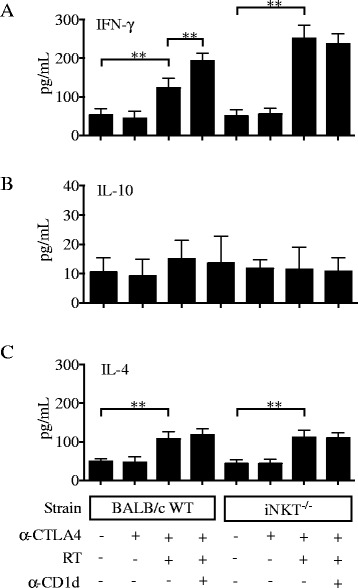
**CD1d blockade enhances systemic IFN-γ response induced by RT + anti-CTLA-4 blockade.** WT or iNKT^−/−^ mice were treated with local tumor radiotherapy in two fractions of 12 Gy given on days 13 and 14 post-tumor inoculation. Mice received anti-CTLA-4 mAb on days 15, 18 and 21. Some mice additionally received anti-CD1d mAb or isotype mAb i.p. on days 3, 7 and 11 post tumor inoculation. Spleen were harvested at day 22 and concentration of **(A)** IFN-γ, **(B)** IL-10 and **(C)** IL-4 were measured in supernatants of PMA + ionomycin stimulated cells. Bars indicate the mean ± SD of 4 mice/group. ^**^ *p* < 0.005.

Next, we investigated whether the improved anti-tumor T cell response seen in the presence of CD1d blocking mAb results in improved tumor response to treatment. To this end, WT 4T1 tumor-bearing mice were treated with RT + anti-CTLA-4 in the presence or absence of CD1d blockade (Figure 4A). Treatment with RT and anti-CTLA-4 was effective at controlling the primary irradiated tumor and extending significantly mice survival compared to control (median survival 41 versus 32.5 days, p < 0.05) (Figure 4B and C), as previously shown [29]. However, no complete tumor regression was achieved and all mice eventually succumbed to metastases. CD1d blockade did not have any effect by itself on tumor growth or survival, but it improved control of the irradiated tumor (p < 0.05) and survival (p < 0.05) of mice treated with RT and anti-CTLA-4 (Figure 4B and C). Complete tumor regression was seen in 25% of the mice and these mice survived long-term and rejected a challenge of 4T1 cells 150 days after the initial inoculum (data not shown). Interestingly, CD1d blockade did not change significantly intra-tumoral levels of TNF-α, IL-10, and IL-4 in mice treated with radiation and anti-CTLA-4 (Figure 5B-D). IL-17 was undetectable in all conditions (data not shown). However, CD1d blockade enhanced significantly the levels of IFNγ induced in tumors by treatment with radiation and anti-CTLA-4 (Figure 5A), suggesting that iNKT cells hinder therapeutically effective Th1 responses in WT mice. Consistently, intra-tumoral levels of IFNγ were high in iNKT^−/−^ mice treated with radiation and anti-CTLA-4 and were not further increased by CD1d blockade. Similarly, IFNγ was produced at significantly higher levels by spleen cells from mice treated with radiation and anti-CTLA-4 upon ex vivo activation in both WT and iNKT^−/−^ mice, and it was further increased by CD1d blockade in WT but not iNKT^−/−^ mice (Figure 6A). Interestingly, while IL-10 levels were low and were not affected by any of the treatments, IL-4 was produced at significantly higher levels by spleen cells of mice treated with RT + anti-CTLA-4 compared to control (Figure 6B and C). However, this effect was independent from iNKT cells since IL-4 levels were similar in WT and iNKT^−/−^ mice and were not enhanced by CD1d blockade in WT mice.

Taken together, these results indicate that iNKT cells can actively suppress the development and/or function of anti-tumor T cells and impair response to anti-CTLA-4 immunotherapy in 4T1 tumor-bearing mice.

### iNKT cells regulate the response of 4T1 tumor-bearing mice to the combination of local radiotherapy and CD137 costimulation

**Figure 7:**
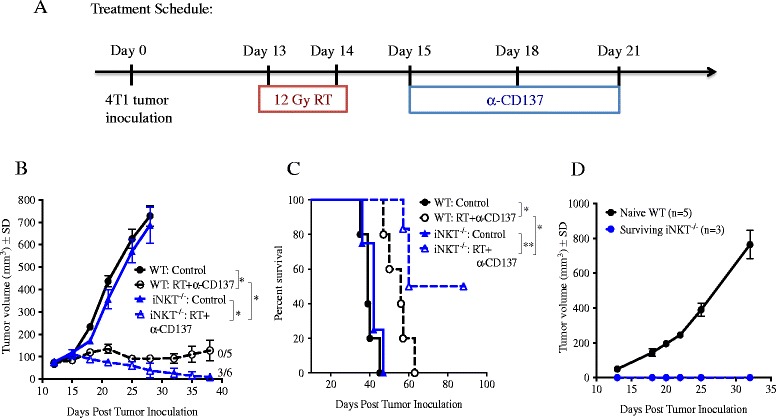
**Response of 4T1 tumor-bearing mice to treatment with local radiotherapy and anti-CD137 mAb is improved in the absence of iNKT cells.** WT or iNKT^−/−^ mice were injected s.c. with 4T1 cells and randomly assigned to treatment groups (N = 5-6 mice/group) on day 13 when tumors became palpable. Local tumor radiotherapy (RT) in two fractions of 12 Gy was given on days 13 and 14 post-tumor inoculation and anti-CD137 mAb on days 15, 18 and 21. **(A)** Treatment schema. **(B)** Tumor growth over time. Fractions indicate the number of mice showing complete tumor regression. **(C)** Kaplan-Meier survival curves. **(D)** Tumor growth in naïve WT mice and iNKT^−/−^ mice that rejected tumors and were challenged on day 120 with 4T1 cells. Data is representative of 2 independent experiments. ^*^ *p* < 0.05.

To determine whether negative regulation by iNKT cells could influence the response to a different immunotherapy, we tested treatment with local radiotherapy in combination with an agonistic anti-CD137 (4-1BB) mAb (Figure 7A). CD137 is a member of the tumor necrosis receptor superfamily that is upregulated shortly after T-cell activation [33]. CD137 ligation delivers a strong survival signal to T-cells, stimulates their effector function and promotes their differentiation into memory cells [34-37]. Importantly, we previously demonstrated that the combination of radiotherapy and CD137 costimulation was significantly more effective than each treatment alone in improving survival of mice with the intracranial GL261 glioma achieving tumor eradication in the majority of mice [38].

**Figure 8:**
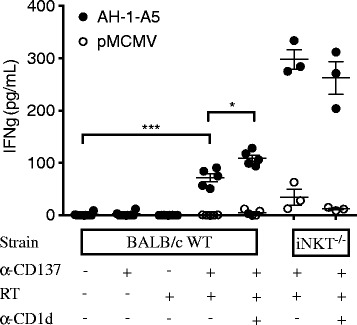
**CD1d blockade enhances production of IFN-γ by tumor-specific cells generated by RT + anti-CD137 co-stimulation.** WT or iNKT^−/−^ mice were treated with local tumor radiotherapy in two fractions of 12 Gy given on days 13 and 14 post-tumor inoculation. Mice received anti-CD137 mAb on days 15, 18 and 21. Some mice additionally received anti-CD1d mAb or isotype control on days 3, 7 and 11 post tumor inoculation. Cells from tumor-draining lymph nodes were collected on day 23 and stimulated with feeder cells pre-loaded with irrelevant peptide pMCMV (open circles) or AH-1-A5 (filled circles). Secreted IFN-γ in the supernatant was measured 48 hours later. Each symbol represents an individual mouse. Bars indicate the mean ± SD of 3–5 mice/group. ^*^ *p* < 0.05, ^***^ *p* < 0.0005.

In both WT and iNKT^−/−^ mice anti-CD137 mAb given as a single agent did not enhance survival compared to control untreated mice (median survival in WT mice: 39 days in control vs 40 days in anti-CD137 group, p = 0.36; iNKT^−/−^ mice: 42 days in control vs 46 days in anti-CD137 group, p = 0.33, Additional file 4: Figure S4). Consistent with previous observations that radiotherapy alone does not inhibit 4T1 lung metastases [29], median survival in WT or iNKT^−/−^ mice given radiotherapy was not enhanced significantly (WT mice: 42 days, p = 0.75 compared to control; iNKT^−/−^ mice: 40 days, p = 0.98 compared to control). In contrast, radiotherapy and anti-CD137 in combination significantly prolonged survival in WT (median survival 56 days, p < 0.05 compared to control) and iNKT^−/−^ (median survival 60 days, p < 0.005 compared to control) mice (Figure 7C). Importantly, no complete tumor regression was achieved in WT mice treated with this combination and all mice eventually succumbed to metastases. In contrast, complete tumor regression and long-term survival was achieved in 50% of iNKT^−/−^ mice treated with radiotherapy + anti-CD137 (Figure 7C). Furthermore, all of the long-term regressors were able to reject a tumorigenic challenge of viable 4T1 cells 120 days after the initial tumor implantation, indicating that they had developed a long-lasting protective immunological memory to the tumor (Figure 7D). To determine if blockade of CD1d in the context of radiotherapy and CD137 costimulation resulted in improved priming of anti-tumor CD8^+^ T cells in dLN of WT mice similar to that observed during anti-CTLA-4 blockade (Figure 3), we measured tumor antigen-specific production of IFNγ by dLN cells. While mice treated with anti-CD137 monotherapy did not show any peptide-specific IFNγ production, treatment with the combination of RT and anti-CD137 induced significant tumor-specific IFNγ production in both WT and iNKT^−/−^ mice (Figure 8). Importantly, the response was markedly higher in iNKT^−/−^ mice and was significantly enhanced by in vivo blockade of CD1d in WT (mean IFN-γ 109.03 ± 13.85 pg/mL in RT + anti-CD137 + anti-CD1d versus 71.58 ± 17.06 pg/mL in RT + anti-CD137, p < 0.05) but not iNKT−/− mice.

Overall, these results indicate that iNKT cells hinder priming of tumor-specific CD8^+^ T cells independent of the immune response modifier used in combination with radiotherapy to treat 4T1 tumor-bearing mice.

## Discussion

The 4T1 mouse mammary carcinoma is considered a model for the aggressive triple negative subtype of human breast cancer [39]. 4T1 cells are highly metastatic and relatively resistant to many immunotherapies once established, including anti-CTLA-4 treatment [29]. We have previously shown that the combination of local radiotherapy with anti-CTLA-4 induces a CD8^+^ T cell-mediated anti-tumor response effective at controlling the irradiated tumor as well as systemic metastases leading to increased survival of 4T1 tumor-bearing mice [29,32,40]. However, cures were rarely seen and most mice eventually succumbed to the tumor. Intriguingly, response to radiotherapy and anti-CTLA-4 was markedly improved in mice lacking iNKT cells, achieving cures in 50% of the mice [25]. The improved response could not be attributed to altered immunogenicity of 4T1 cells in iNKT^−/−^ mice and could not be recapitulated in WT mice by administration of α-GalCer, a potent activator of iNKT cells anti-tumor activity in many tumor models [12,13,41-44]. Here we show that the improved response of 4T1 tumor-bearing mice seen in the absence of iNKT cells is not unique to anti-CTLA-4-based immunotherapy. Co-stimulation of T cells by anti-CD137 in combination with radiotherapy was similarly more effective in iNKT^−/−^ than WT mice and resulted in protective anti-tumor recall responses. These data further establish a negative regulatory role of iNKT cells in the 4T1 tumor model.

To understand the mechanisms of immune regulation by iNKT cells we examined DC, which are largely CD1d^+^, and are the APCs required for cross-presentation of tumor antigens to T cells [45,46]. Surprisingly, there was a reduction in the number of DCs present in the tumor and dLN of WT mice compared to iNKT^−/−^ mice (Figure 1). Downregulation of surface CD11c molecules has been reported in TLR-activated mouse DCs [47]. We did not see any difference by flow cytometry in the levels of CD11c in DCs isolated from non-draining LN, dLN and tumors (not shown), suggesting that CD11c downregulation induced by activation of DCs in the tumor of WT mice is not likely to account for locoregional reduction of DCs. Moreover, blocking the interaction of iNKT cells with DCs with anti-CD1d mAb led to an increase in both CD8α + (immunostimulatory) and CD8α- DCs in WT mice without affecting their activation state as measured by expression of costimulatory molecules (Figure 2). Overall, data indicate that iNKT cells regulate DCs numbers in the tumor and dLN.

Clearance of APC has been proposed as a mechanism to prevent unrestrained T-cell activation and proliferation, which is beneficial in preventing systemic autoimmunity [48] but detrimental for anti-tumor responses [49]. Several immune cells, including Tregs [26], NK cells [50] and CD8^+^ CTLs [49,51], have been implicated in direct elimination of DCs. We were unable to isolate tumor-infiltrating iNKT in sufficient quantities to test their ability to kill DCs ex vivo, but human iNKT cells have been shown to kill DCs in vitro [52], and it is possible that a similar mechanism is responsible for DC reduction in 4T1 tumor-bearing mice.

Blockade of CD1d in WT mice increased not only locoregional DC numbers, but also the priming of tumor-specific CD8 T cells in dLN and the overall tumor response to treatment with radiotherapy and anti-CTLA-4 mAb, suggesting that temporarily “disabling” iNKT cells was sufficient to enhance Th1 type anti-tumor immunity. The increased production of IFNγ in the tumor and spleen of WT mice treated with RT + anti-CTLA-4 in the presence of CD1d blockade supports this interpretation.

The overall better anti-tumor immunity and the high levels of IFNγ seen in treated iNKT^−/−^ mice could be due to a larger population of immunostimulatory DC presenting tumor-derived antigens, but may also reflect skewing of the immune system that develops in BALB/c mice in the absence of iNKT cells towards Th1 rather than Th2 immunity [53]. IL-4 producing iNKT cells (NKT2) were recently described and shown to be the dominant iNKT subset in the thymus of BALB/c mice, and were implicated in determining the Th2 dominance of this mouse strain [53]. Among tumor-infiltrating iNKT cells, we found a larger percentage (6%) that expressed markers (CD27 + CD122-) shown to define iNKT2 subset, while only 0.1% had markers of iNKT1 (CD27 + CD122+) cells (Additional file 5: Figure S5). However, the majority was CD27-CD122-, a phenotype associated with IL-17 production in the thymus [53]. Since IL-17 was undetectable in 4T1 tumors, defining the functional differentiation of iNKT cells in the tumor microenvironment may require different markers than in the thymus. Importantly, comparison between IL-4 levels produced in the tumor and spleen of tumor-bearing WT and iNKT^−/−^ mice did not reveal significant differences consistent with differential Th2 polarization. Intratumoral IL-4 levels were higher in treated WT than iNKT^−/−^ mice, but unlike IFNγ, IL-4 levels were not modulated by CD1d blockade (Figure 5D). In the spleen IL-4 was increased similarly in treated WT and iNKT^−/−^ mice (Figure 6C).

iNKT cell role in promoting immune tolerance has been established in several experimental conditions, including autoimmune disease models, organ transplant and tolerance to oral antigens [23]. While multiple mechanisms have been suggested to play a role, to our knowledge control of DCs numbers by iNKT cells has not been previously documented in immune tolerance models or in the setting of cancer [23,24,54,55]. However, evidence that human iNKT cells can kill DCs in vitro [52], suggests that such mechanism could contribute to immunosuppression in some tumors and be responsible, at least in part, for the lack of clinical activity of α-GalCer in cancer patients [56,57]. Consistent with this hypothesis, administration of α-GalCer to 4T1 tumor-bearing mice at day 15 post-tumor inoculation failed to show any effect by itself or in combination with immunotherapy [25]. By this time, DCs are low in tumors and dLN (Figure 1), and the remaining DCs may be insufficient to present α-GalCer and/or may have acquired a tolerogenic phenotype.

The identity of the CD1d ligand(s) presented by DCs to iNKT cells in 4T1 tumors and dLN is unknown. The endogenous lipids presented by CD1d in steady state conditions are likely to be non-antigenic or elicit tolerance. However, metabolic changes in the tumor may lead to altered lipid biosynthesis and generation of qualitatively or quantitatively modified CD1d ligands capable of triggering a different functional response of iNKT cells, further modulated by the inflammatory environment [23]. Improved understanding of the relationship between lipid metabolism in cancer and immune regulation will pave the way to the design of more effective immunotherapies that can enlist the power of iNKT cells to elicit strong anti-tumor immunity [58].

## Conclusions

In this study we show that local reduction of DCs mediated by iNKT cells regulates the response to immunotherapy in a mouse model of aggressive breast cancer. The elimination of APC that cross-present tumor-derived antigens in dLNs was shown previously to be one mechanism of immunosuppression mediated by Tregs [26]. However, iNKT cells were believed to consistently perform anti-tumor activities, while immunosuppressive functions were ascribed to the non-invariant subset of NKT cells [59]. Here we demonstrate for the first time a novel mechanism of immune escape mediated by iNKT cells.

These results have important implications for the design of immunotherapies targeting iNKT cells (reviewed in [58]). CD1d blockade, or the iNKT-depleting antibody NKTT120 recently developed for inflammatory diseases [60], could have a role in treatment of cancers in which iNKT acquire immunosuppressive functions.

## Methods

### Cells, antibodies and reagents

4T1 is a BALB/c-derived mammary carcinoma that is highly metastatic and mimics the behavior of triple negative human breast cancer [39,61]. 4T1 cells were grown in complete medium consisting of DMEM (Invitrogen) supplemented with 2 mol/L L-glutamine, 100 U/mL penicillin, 100 μg/mL streptomycin, 2.5 × 10^−5^ mol/L 2-mercaptoethanol and 10% fetal bovine serum (BioWest). Cells were confirmed to be free of mycoplasma contamination using a mycoplasma detection kit (Sigma). DN32.D3 hybridoma cells, kindly provided by Masaki Terabe from the NCI Branch of the National Institutes of Health, were grown in supplemented RPMI (Invitrogen) [62]. Anti-CTLA-4 hamster monoclonal antibody (Clone 9H10) and anti-CD1d rat monoclonal antibody (Clone 20H2) were purchased from BioXCell (West Lebanon, NH). Control hamster IgG (isotype control for anti-CTLA-4) was purchased from Jackson Immunoresearch Laboratories. Control rat IgG1 (isotype control for anti-CD1d) was purchased from BioXCell. A rat IgG2a mAb against mouse CD137 (BMS-469492, clone 1D8) was provided for these studies by Bristol-Myers Squibb (Princeton, NJ).

### Animals

Six to eight-week old WT BALB/c mice were purchased from Taconic. iNKT^−/−^ (Vα14Jα18-deficient) mice [27] in the BALB/c background obtained from M. Taniguchi were bred at Berg Animal Facility at NYU School of Medicine and used between 6 to 8 weeks of age. All experiments were approved by the Institutional Animal Care and Use Committee of NYU Langone Medical Center.

### In vivo treatment

Mice were inoculated subcutaneously (s.c.) with 5 × 10^4^ 4T1 cells and randomly assigned to treatment groups 13 days later when tumors reached an average diameter of 5 mm. Radiotherapy was given as previously described [25] with some modifications. Lightly anesthetized mice were positioned on a dedicated plexiglass tray with only the tumor area exposed to radiation while the rest of the body was protected by lead shielding. Radiotherapy was given using the Small Animal Radiation Research Platform (SARRP) (Gulmay Medical, Suwanee, GA) [63] in two doses of 12 Gy each on days 13 and 14 post tumor inoculation. Control hamster IgG and anti-CTLA-4 or anti-CD137/4-1BB mAbs were given at 200 μg i.p. at 1, 4 and 7 days after completion of radiotherapy. To block CD1d in vivo mice were given three doses of anti-CD1d mAb (Clone 20H2) at 100 μg i.p. on days 3, 7 and 11 post tumor inoculation. Tumors were measured every 2–3 days until death or sacrifice when tumor dimensions exceeded 5% of body weight or if animals showed signs of significant pain or distress due to metastatic disease. In some experiments, mice that eradicated the tumor after treatment and remained tumor-free for at least 120 days were inoculated in the contralateral flank with 4T1 cells and tumor growth monitored. A group of naïve mice was similarly challenged with 4T1 cells as control.

### Immunohistochemistry

4T1 tumors and dLNs were harvested on indicated days, fixed in freshly prepared 4% paraformaldehyde for 1 hr at 4°C and incubated overnight in 30% sucrose. Tumors were then frozen in optimum cutting temperature (OCT) medium and stored at −80°C. Sections (5 μm) were treated with 3% H_2_O_2_ in PBS for 30 minutes in order to eliminate endogenous peroxide activity. After blocking in 4% hamster serum for an hour and three washes with PBS, anti-mouse CD11c eFluor 615 (eBioscience, San Diego, CA) was applied at a 1/100 dilution in 1% BSA for 1 hour, followed by three washes and incubation in HRP-conjugated streptavidin (Jackson ImmunoResearch, West Grove, PA) at a 1/2,000 dilution in 1% BSA for 30 minutes. To amplify the signal, slides were subject to two rounds of biotin-tyramide amplification (PerkinElmer, Melville, NY) using manufacturer’s suggested protocol. The slides were counterstained with DAPI, mounted with Vectashield and images obtained using a Nikon Eclipse 800 deconvolution microscope. Positive cells were counted from at least 3 randomly selected fields (20× magnification).

### Flow cytometry

Lymph nodes and minced tumors were digested using a cocktail of 1.67 Wünsch U/mL Liberase TL (Roche) and 0.2 mg/mL DNAse (Roche) as previously described [40,64]. After lysis of red blood cells single cell suspensions were filtered through a 40-μm nylon cell strainer. Live cells were distinguished using Fixable Viability Dye eFluor 660 (eBioscience) prior to flow staining. All samples were incubated with anti-mouse CD16/32 (Fc block) for 10 minutes followed by staining for various surface markers for 30 minutes at 4°C. The following antibodies were used (all purchased from eBiosciences): PE-Cy7-conjugated anti-mouse CD11c, Alexa Fluor 700-conjugated anti-mouse CD86, PE-conjugated anti-mouse CD80, PE-conjugated anti-mouse CD1d, PE-conjugated anti-mouse CD70, FITC-conjugated anti-mouse CD40 and Alexa Fluor 700-conjugated anti-mouse MHC Class I (H-2Kd/Dd), FITC-conjugated anti-mouse CD27, PECy7-conjugated anti-mouse CD3ε, APC-conjugated anti-mouse CD122, Alexa Fluor 700-conjugated anti-mouse CD4. iNKT cells were identified by staining with mouse CD1d/PBS-57 or CD1d/unloaded tetramers (NIH Tetramer Core Facility) for 30 minutes at room temperature [65]. All samples were analyzed using a BD LSR II flow cytometer and FlowJo version 8.7.5 (Tree Star).

### Ex vivo cytokine production

Single cell suspensions (3 × 10^5^ per well) from draining lymph nodes of 4T1 tumor-bearing WT or iNKT^−/−^ mice were cultured in complete T-cell medium in 48-well tissue culture plates with irradiated (12 Gy) feeder cells (3 × 10^6^) prepared from the spleen of healthy BALB/c mice pre-loaded with tumor-derived peptides (AH-1-A5) or an H-2L^d^-binding irrelevant peptide (pMCMV) used at a final concentration of 1 μg/mL. Peptide sequences are SPSYAYHQF (AH-1-A5) [66], and YPHFMPTNL (pMCMV) [66]. After 48 hours, supernatants were collected and stored at −80°C. Secreted IFN-γ was measured from duplicate wells using Flowcytomix kit (eBioscience, San Diego, CA). Background IFN-γ production from supernatants of cells cultured in complete T-cell media alone was subtracted. Spleen cell suspensions (10^6^) from 4T1 tumor-bearing mice were cultured in a 24-well tissue culture plate and stimulated with PMA + ionomycin Cell Stimulation Cocktail Mix (eBioscience) for 48 hours.

### Intratumoral cytokines

Freshly harvested tumors were immediately frozen in liquid nitrogen and stored at −80°C. To isolate total protein, tissues were thawed on ice, minced and homogenized in lysis buffer (10 mM Tris–HCl pH8.0, 150 mM NaCl, 1%NP-40, 10% glycerol, 5 mM EDTA) and protease inhibitor cocktail (Roche). Total volume (μL) of lysis buffer used was equal to 5× weight of tumor tissue (mg). Lysates were sonicated in 4°C using a 7-minute regimen of 30s intermittent on and off. Samples were spun at 14,000 rpm for 15 minutes at 4°C, supernatants collected and protein concentrations determined using Bradford assay. Samples were stored at −80°C. Cytokines were measured from duplicates wells using multi-analyte Flowcytomix kit.

### Statistical analysis

Random coefficients regression (RCR) was used to assess the effect of treatment on tumor growth. The dependent variable was the natural log of tumor volume at all available times; log volumes were used since the change in log volume over the course of follow-up was well approximated as linear within each treatment arm. Log rank tests were used to compare treatment arms in terms of survival. The Kaplan-Meier method was used to estimate median survival times and the log cumulative hazard transformation was used to derive 95% confidence limits for median survival in each arm. All reported p values are two-sided and statistical significance defined as p < 0.05. SAS 9.3 (SAS Institute, Cary, NC) was used for all computations.

## Additional files

Additional file 1: Figure S1
**Anti-CD1d mAb blocks cytokine production by DN32.D3 NKT-like hybridoma cells stimulated with α-GalCer-loaded DCs.** CD11c^+^ bone-marrow derived dendritic cells (BMDC) from healthy BALB/c mice were plated in 24-well tissue culture plate (2×10^4^/well) and incubated with 100 ng/mL α-GalCer overnight, washed extensively and incubated overnight with fresh media containing anti-CD1d at indicated final concentrations. Cells were washed again before the addition of DN32.D3 hybridoma cells (10^5^/well). Supernatants were collected three days later and cytokines measured. Additional file 2: Figure S2
**Anti-CD1d mAb does not deplete CD1d-expressing cells in vivo.** Healthy WT mice (N = 4/group) were given isotype or anti-CD1d mAb i.p. (200 μg/mouse) every 4 days for a total of 3 doses. Two days after the last dose, mice were euthanized and single cell suspensions from digested spleens collected for flow staining of **(A)** DC, **(B)** myeloid cells, **(C)** B-cells and **(D)** macrophage populations. Additional file 3: Figure S3
**Anti-CD1d mAb does not induce stimulation of CD1d-expressing APCs.** Splenocytes from naïve WT or iNKT^−/−^ mice (n = 4/group) were stimulated in vitro with 10 μg/mL α-CD1d or isotype control mAbs for 48 hours. As a positive control, splenocytes were stimulated with LPS (1 μg/mL). Supernatants were collected and measured for secreted **(A)** IL-10, **(B)** IFN-γ, **(C)** IL-12p70 and **(D)** IL-4. Additional file 4: Figure S4
**Local tumor radiotherapy and CD137 co-stimulation used alone are ineffective in prolonging survival of 4T1 tumor-bearing mice.** WT and iNKT^−/−^ mice were injected s.c. with 4T1 cells and randomly assigned to treatment groups (n = 5-6/group) on day 13 when tumors became palpable. Local tumor radiotherapy (RT) was given in two fractions of 12 Gy on days 13 and 14 post-tumor inoculation. Mice received anti-CD137 mAb on days 15, 18 and 21. Kaplan-Meier curves were generated from survival data of each mouse calculated as time (days) from date of inoculation until death or euthanasia. Median survival (days) in each treatment group is indicated in parenthesis. Data is representative of 2 independent experiments. Additional file 5: Figure S5
**iNKT subset compartments in 4T1 tumors and tumor-draining lymph nodes.** WT mice were injected s.c. with 4T1 cells. On day 15, tumor samples from three mice were pooled and digested to obtain single cell suspensions. **(A)** Intratumoral iNKT cells were identified using mouse CD1d/PBS-57 tetramers. Expression of surface CD122 and CD27 antigens were assessed on CD3+ CD1d/PBS-57 tetramer + populations to identify iNKT1 (CD27 + CD122+) and iNKT2 (CD27-CD122+) subsets. **(B)** The relative abundance of iNKT cells in the tumor and draining lymph nodes is shown as a percentage of total CD3 cells. **(C)** The relative abundance of iNKT1 (white bars) and iNKT2 (black bars) subsets is shown as a percentage of total CD3+ CD1d/PBS-57 tetramer + cells. Bars indicate the mean ± SD of three samples. ^**^ *p* < 0.005.

## Abbreviations

α-GalCer: Alpha galactosylceramide
APC: Antigen presenting cells
BMDC: Bone marrow-derived dendritic cells
CD: Cluster of differentiation
CTL: Cytotoxic T lymphocytes
DCs: Dendritic cells
dLN: Draining lymph node
Gy: Gray
IFN-γ: Interferon gamma
IHC: Immunohistochemistry
iNKT^−/−^: Invariant natural killer T cell-deficient
i.p.: Intraperitoneal
mAb: Monoclonal antibody
MHC: Major histocompatibility complex
ndLN: Non-draining lymph node
NK: Natural killer
PBS: Phosphate buffered saline
PMA: Phorbol 12-myristate 13-acetate
RT: Radiotherapy
s.c.: Subcutaneous
TAA: Tumor-associated antigens
Th1: T-helper type 1
Th2: T-helper type 2
Tregs: Regulatory T cells
WT: Wild type
