# Supplementary figures and images for: Invariant natural killer T cells regulate anti-tumor immunity by controlling the population of dendritic cells in tumor and draining lymph nodes
Source: J Immunother Cancer. 2014 Oct 14;2:37. doi: 10.1186/s40425-014-0037-x (PMC4206765; doi:10.1186/s40425-014-0037-x)

## Slide 1
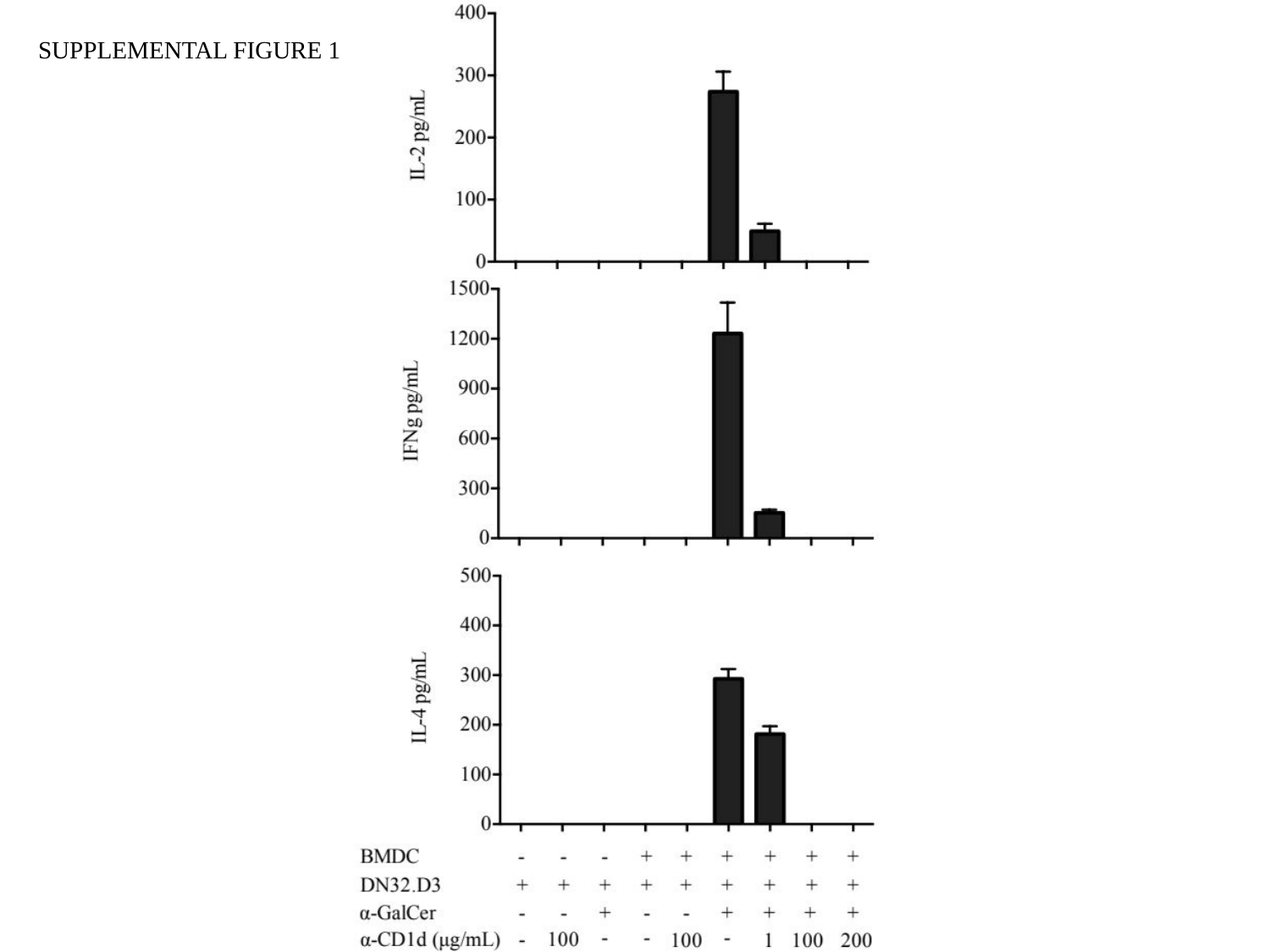

SUPPLEMENTAL FIGURE 1

Supplement: Additional file 1: Figure S1 — Anti-CD1d mAb blocks cytokine production by DN32.D3 NKT-like hybridoma cells stimulated with α-GalCer-loaded DCs. CD11c+ bone-marrow derived dendritic cells (BMDC) from healthy BALB/c mice were plated in 24-well tissue culture plate (2×10^4^/well) and incubated with 100 ng/mL α-GalCer overnight, washed extensively and incubated overnight with fresh media containing anti-CD1d at indicated final concentrations. Cells were washed again before the addition of DN32.D3 hybridoma cells (105/well). Supernatants were collected three days later and cytokines measured. [file 40425_2014_37_MOESM1_ESM.pptx]

## Slide 1
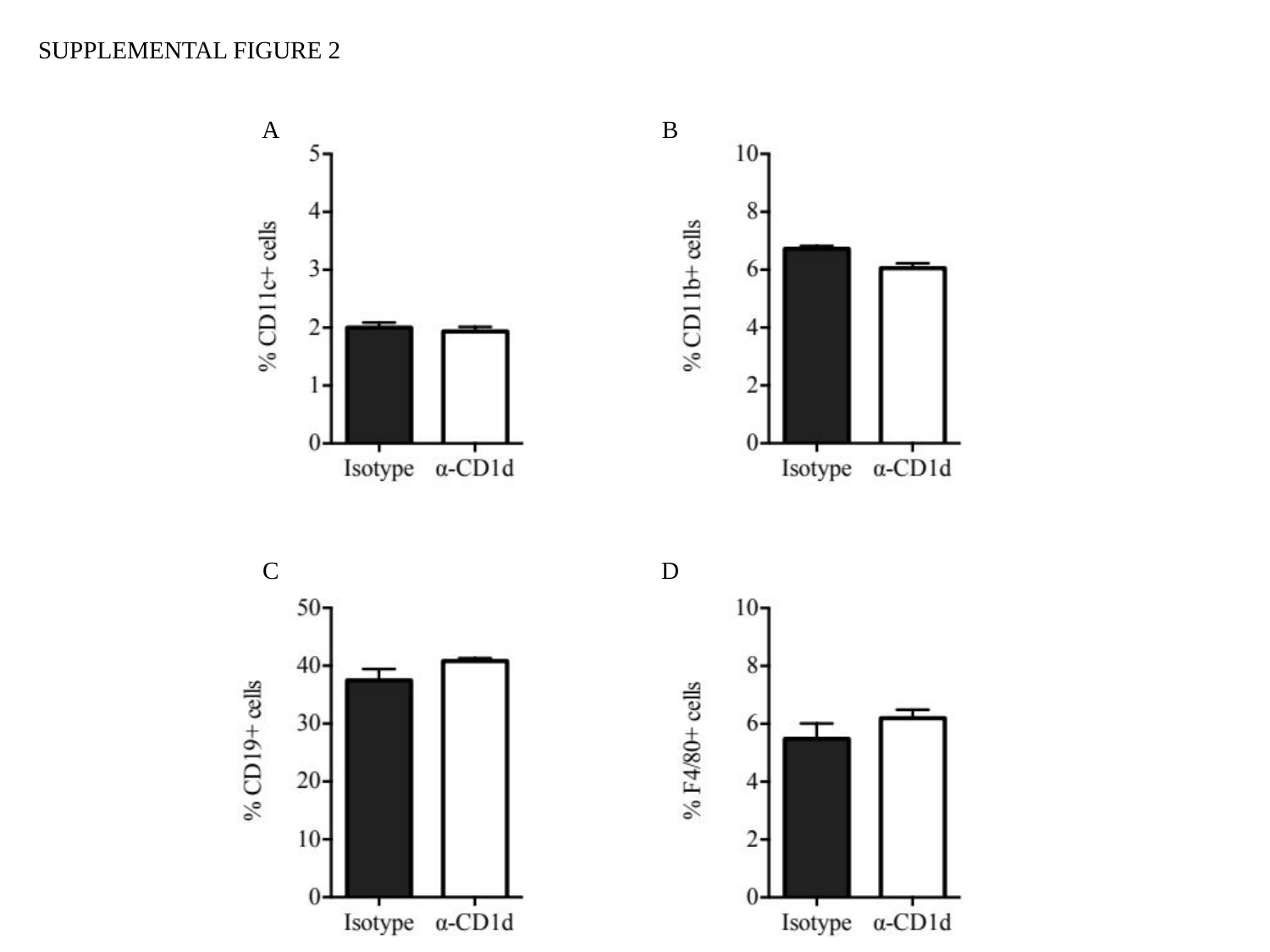

SUPPLEMENTAL FIGURE 2
A
B
C
D

Supplement: Additional file 2: Figure S2 — Anti-CD1d mAb does not deplete CD1d-expressing cells in vivo. Healthy WT mice (N = 4/group) were given isotype or anti-CD1d mAb i.p. (200 μg/mouse) every 4 days for a total of 3 doses. Two days after the last dose, mice were euthanized and single cell suspensions from digested spleens collected for flow staining of (A) DC, (B) myeloid cells, (C) B-cells and (D) macrophage populations. [file 40425_2014_37_MOESM2_ESM.pptx]

## Slide 1
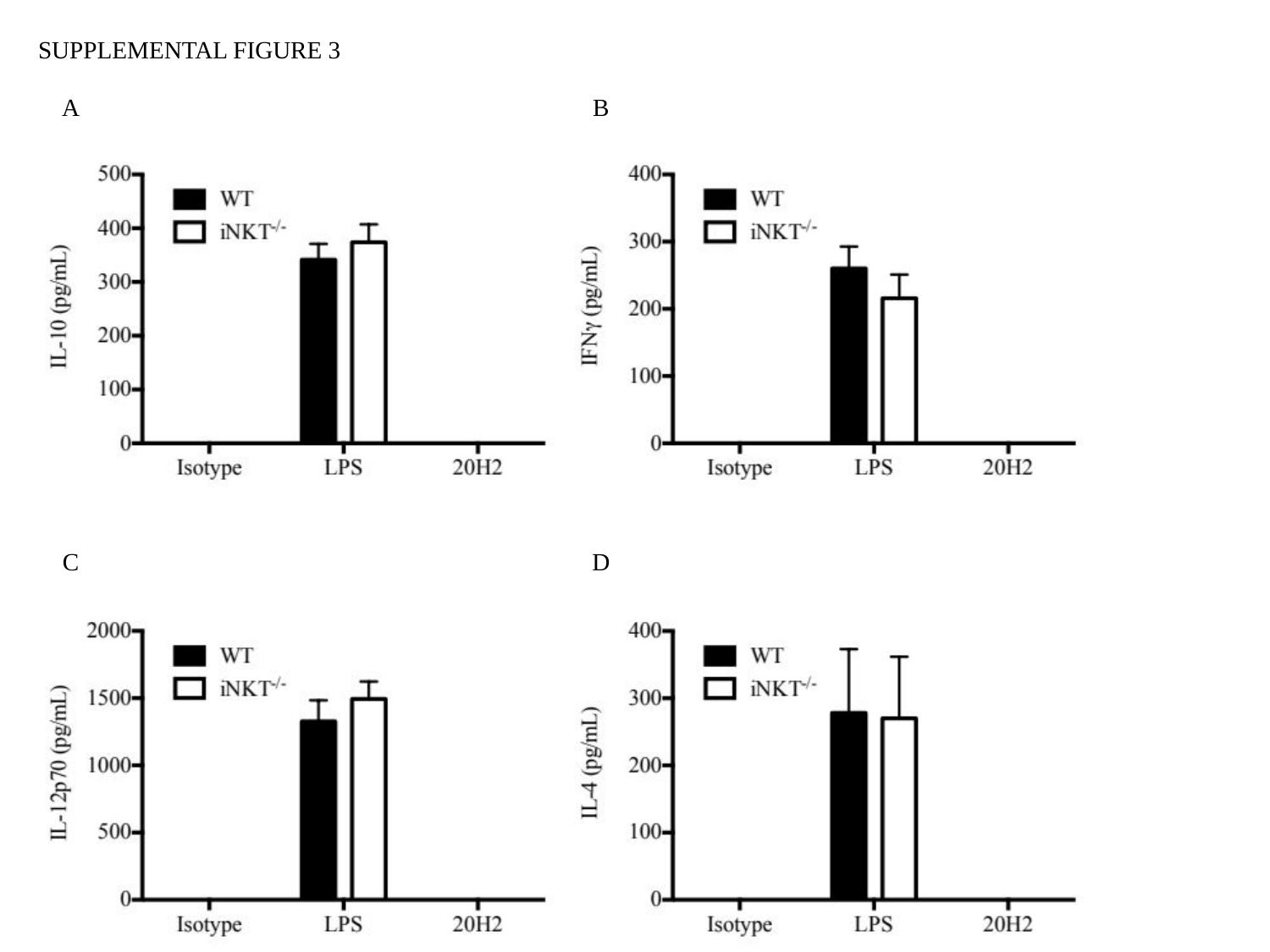

SUPPLEMENTAL FIGURE 3
B
A
C
D

Supplement: Additional file 3: Figure S3 — Anti-CD1d mAb does not induce stimulation of CD1d-expressing APCs. Splenocytes from naïve WT or iNKT−/− mice (n = 4/group) were stimulated in vitro with 10 μg/mL α-CD1d or isotype control mAbs for 48 hours. As a positive control, splenocytes were stimulated with LPS (1 μg/mL). Supernatants were collected and measured for secreted (A) IL-10, (B) IFN-γ, (C) IL-12p70 and (D) IL-4. [file 40425_2014_37_MOESM3_ESM.pptx]

## Slide 1
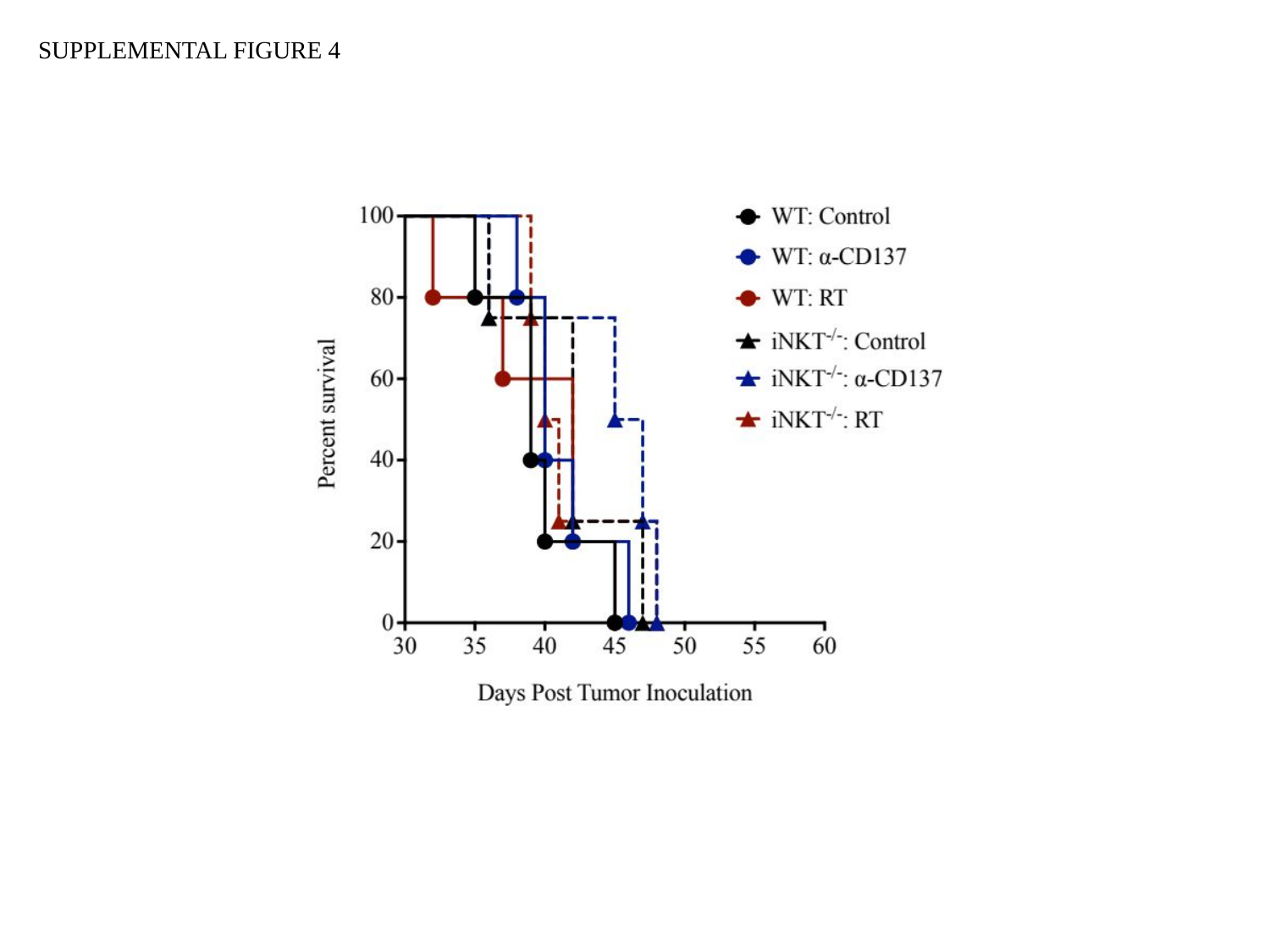

SUPPLEMENTAL FIGURE 4

Supplement: Additional file 4: Figure S4 — Local tumor radiotherapy and CD137 co-stimulation used alone are ineffective in prolonging survival of 4T1 tumor-bearing mice. WT and iNKT−/− mice were injected s.c. with 4T1 cells and randomly assigned to treatment groups (n = 5-6/group) on day 13 when tumors became palpable. Local tumor radiotherapy (RT) was given in two fractions of 12 Gy on days 13 and 14 post-tumor inoculation. Mice received anti-CD137 mAb on days 15, 18 and 21. Kaplan-Meier curves were generated from survival data of each mouse calculated as time (days) from date of inoculation until death or euthanasia. Median survival (days) in each treatment group is indicated in parenthesis. Data is representative of 2 independent experiments. [file 40425_2014_37_MOESM4_ESM.pptx]

## Slide 1
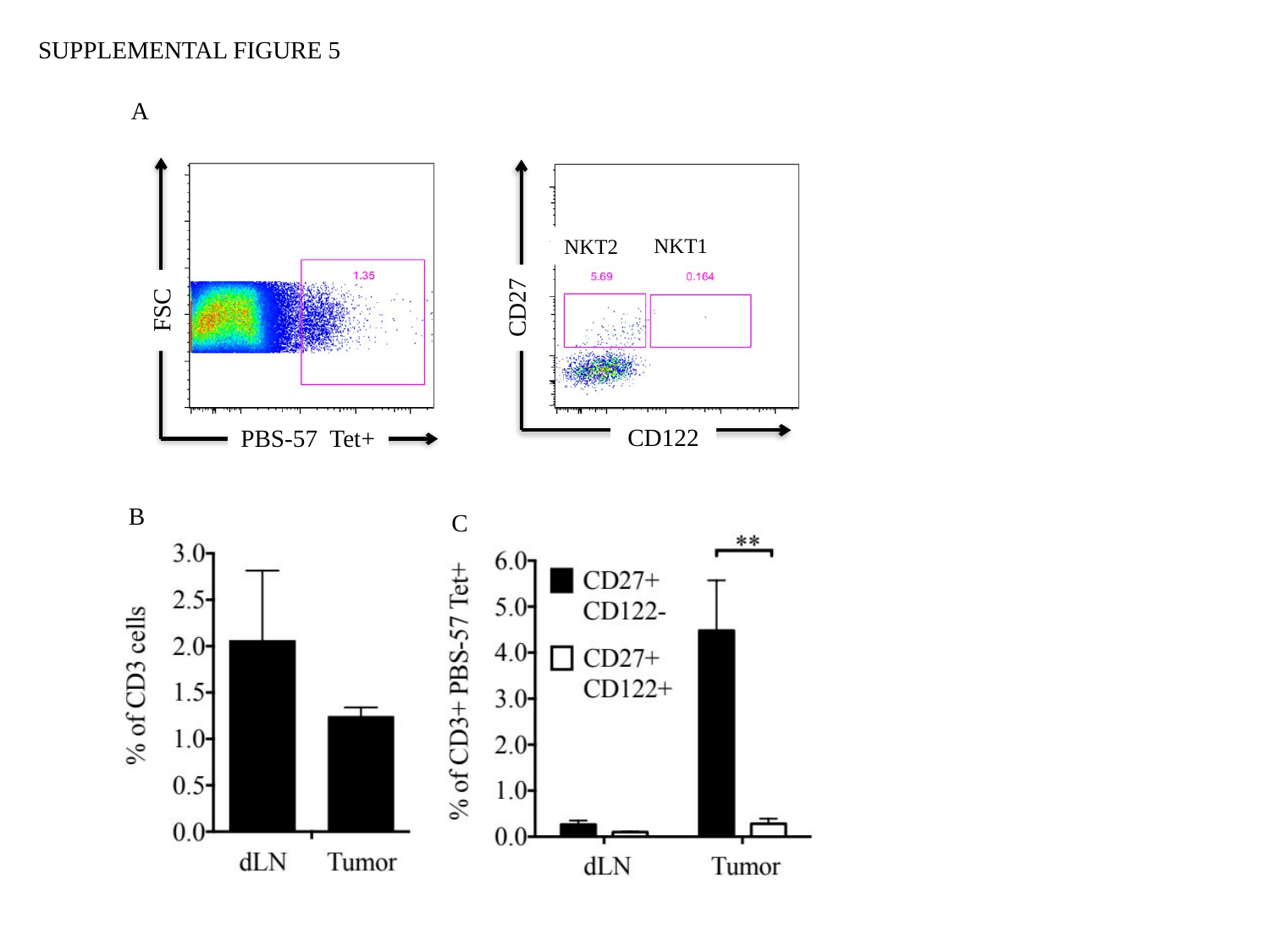

SUPPLEMENTAL FIGURE 5
A
NKT1
NKT2
CD27
FSC
CD122
PBS-57 Tet+
B
C

Supplement: Additional file 5: Figure S5 — iNKT subset compartments in 4T1 tumors and tumor-draining lymph nodes. WT mice were injected s.c. with 4T1 cells. On day 15, tumor samples from three mice were pooled and digested to obtain single cell suspensions. (A) Intratumoral iNKT cells were identified using mouse CD1d/PBS-57 tetramers. Expression of surface CD122 and CD27 antigens were assessed on CD3+ CD1d/PBS-57 tetramer + populations to identify iNKT1 (CD27 + CD122+) and iNKT2 (CD27-CD122+) subsets. (B) The relative abundance of iNKT cells in the tumor and draining lymph nodes is shown as a percentage of total CD3 cells. (C) The relative abundance of iNKT1 (white bars) and iNKT2 (black bars) subsets is shown as a percentage of total CD3+ CD1d/PBS-57 tetramer + cells. Bars indicate the mean ± SD of three samples. ** p < 0.005. [file 40425_2014_37_MOESM5_ESM.pptx]
